# Impact of the Patient-Driven Groupings Model on Rehabilitation Services in Post-Acute Home Health Care

**DOI:** 10.1093/geroni/igaf122.285

**Published:** 2025-12-31

**Authors:** Tracy Mroz, Arati Dahal, Rachel Prusynski, Debra Saliba, Natalie Leland

**Affiliations:** University of Washington School of Medicine, Seattle, Washington, United States; University of Washington, Seattle, Washington, United States; University of Washington, Seattle, Washington, United States; University of California, Los Angeles, Los Angeles, California, United States; University of Pittsburgh, Pittsburgh, Pennsylvania, United States

## Abstract

Home health (HH) agencies offer skilled nursing and rehabilitation (physical therapy [PT], occupational therapy [OT], and speech language pathology [SLP]) services to facilitate patients’ transition back home following hospitalization. Implemented in 2020, the Patient-Driven Groupings Model (PDGM) removed previous incentives to provide rehabilitation visits, raising concerns about reduced access to these services. Using 100% Traditional Medicare HH claims and assessment data from 2018-2021, we examined changes in receipt of rehabilitation services following PDGM implementation. We modelled odds of receiving any PT, OT, and ST visits across 2,657,967 post-acute HH stays using logistic regression. We modelled number of PT, OT, and ST visits among those who received any visits using negative binomial regression. All models accounted for clustering of stays within agencies and controlled for patient sociodemographic and clinical characteristics, length of stay, agency characteristics, and county-level COVID-19 case rates as the pandemic emerged nearly simultaneously with PDGM implementation. While the adjusted odds of receiving PT, OT, and SLP services during post-acute HH stays were not significantly lower under PDGM compared to before PDGM, the number of visits received decreased significantly for PT (aIRR: 0.86; 95%CI: 0.85,0.86), OT (aIRR: 0.82; 95%CI: 0.82,0.83), and SLP (aIRR: 0.76, 95%CI: 0.75,0.77). This reduction on average equates to about one fewer visit each of PT, OT, and SLP per post-acute HH stay. Decreases in PT, OT, and SLP visits under PDGM may lead to worse rehabilitation-related outcomes for post-acute HH patients. Additional examination of the impact of reduced rehabilitation on patient outcomes is urgently needed.

